# Advances in electrochemical biosensors employing carbon-based electrodes for detection of biomarkers in diabetes mellitus

**DOI:** 10.5599/admet.2361

**Published:** 2024-07-25

**Authors:** Serly Zuliska, Iman Permana Maksum, Yasuaki Einaga, Grandprix Thomreys Marth Kadja, Irkham Irkham

**Affiliations:** 1 Department of Chemistry, Faculty of Mathematics and Natural Sciences, Padjadjaran University, Bandung 40173, Indonesia; 2 Department of Chemistry, Keio University, 3-14-1 Hiyoshi, Yokohama, 223-8522, Japan; 3 Division of Inorganic and Physical Chemistry, Faculty of Mathematics and Natural Sciences, Institut Teknologi Bandung, Jl. Ganesha no. 10, Bandung 40132, Indonesia; 4 Research Center for Nanosciences and Nanotechnology, Institut Teknologi Bandung, Jl. Ganesha no. 10, Bandung 40132, Indonesia

**Keywords:** Biomarker detection, glucose sensing, glycated haemoglobin, novel biomarkers, point-of-care diagnostics, carbon electrodes

## Abstract

**Background and purpose:**

The increase in diabetes cases has become a major concern in the healthcare sector, necessitating the development of efficient and minimal diagnostic methods. This study aims to provide a comprehensive examination of electrochemical biosensors for detecting diabetes mellitus biomarkers, with a special focus on the utilization of carbon-based electrodes.

**Review approach:**

A detailed analysis of electrochemical biosensors incorporating various carbon electrodes, including screen-printed carbon electrodes, glassy carbon electrodes, and carbon paste electrodes, is presented. The advantages of carbon-based electrodes in biosensor design are highlighted. The review covers the detection of several key diabetes biomarkers, such as glucose, glycated hemoglobin (HbA1c), glycated human serum albumin (GHSA), insulin, and novel biomarkers.

**Key results:**

Recent developments in electrochemical biosensor technology over the last decade are summarized, emphasizing their potential in clinical applications, particularly in point-of-care settings. The utilization of carbon-based electrodes in biosensors is shown to offer significant advantages, including enhanced sensitivity, selectivity, and cost-effectiveness.

**Conclusion:**

This review underscores the importance of carbon-based electrodes in the design of electrochemical biosensors and raises awareness for the detection of novel biomarkers for more specific and personalized diabetes mellitus cases. The advancements in this field highlight the potential of these biosensors in future clinical applications, especially in point-of-care diagnostics.

## Introduction

Diabetes mellitus (DM) is a chronic metabolic condition marked by elevated blood glucose levels [1] resulting from impaired insulin secretion, insulin action, or both [2]. The International Diabetes Federation (IDF) reports that in 2021 [3], the death rate caused by DM reached 6.7 million individuals aged 20-79 years. About 44.7% of the world's diabetes population were living with undiagnosed diabetes. Therefore, DM is also called the silent killer disease because of the unawareness of having diabetes by the person with diabetes and when it is known that it has severe complications, making treatment difficult.

Biological markers (biomarkers) are objective indicators that can be used to interpret normal biological processes, pathologies, or responses to certain interventions, including hyperglycemia [4]. Various biomarkers have been used to detect diabetes mellitus, such as glucose, HbA1c, GHSA, and insulin [5], with various methods developed, such as ion exchange chromatography [6,7], affinity chromatography [8,9], capillary electrophoresis [10-13], enzymatic assay [14,15], and immunoassay [16-18]. Methods that use large instruments are not suitable for on-site monitoring, and they have high costs and take a long time to obtain results [19]. Among the different methods, the electrochemical biosensor method has attracted much attention as a disease detection tool because it can produce rapid analysis, use small samples, portability, and miniaturization of the system. Besides, the use of biological compounds as target receptors makes it a more specific detection method with greater sensitivity than electrochemical sensors [20].

The fundamental principle of sensing in electrochemical biosensors is based on the oxidation and reduction reactions of analytes occurring on the surface of the working electrode [21,22]. The working electrode is an important factor affecting sensor performance [23]. Various materials have been developed for use in electrochemical sensors, such as metal materials, metal-organic frameworks, hybrid materials, organic polymers, and carbon-based materials [24]. Carbon-based materials are currently receiving significant attention as working electrodes in the preparation of electrochemical sensors or biosensors due to their wide potential range, non-toxicity, relatively low cost, good electrical conductivity, ease of surface modification, and high electrochemical activity for various redox reactions [24-26]. The sensitivity, specificity, and other capabilities of carbon-based electrodes can be improved or established through modification of the electrode surface using unique materials and characteristics in typical electrochemical systems [27].

Differences in electrode fabrication and modification affect the performance of electrochemical biosensors. Modification with materials such as nanomaterials or polymer coatings can also provide greater availability of active sites for electrochemical reactions and facilitate more efficient immobilisation of bioreceptors [28,29]. The process typically begins with cleaning and chemically activating the sensor surface to introduce functional groups like carboxyl, amine, or hydroxyl groups, which can interact with the polymer. A polymer solution, often comprising biocompatible polymers like polyethylene glycol (PEG), polyvinyl alcohol (PVA), or polypyrrole, is then applied to the activated surface through techniques such as dip-coating, spin-coating, or layer-by-layer assembly [30,31]. The polymer adheres to the surface, forming a stable, uniform layer, and may undergo cross-linking to enhance stability. The polymer layer contains functional groups that facilitate the covalent bonding of bioreceptors. For instance, a polymer with carboxyl groups can react with amine groups on proteins through chemical reactions like EDC/NHS (1-Ethyl-3-(3-dimethylaminopropyl)carbodiimide/N-hydroxysuccinimide) coupling. This covalent bonding ensures a robust attachment of bioreceptors [32], although physical adsorption through hydrophobic interactions, van der Waals forces, or hydrogen bonding can also be employed for less stable applications. Meanwhile, nanoparticles (*e.g.*, gold, silver, silica) are functionalized with reactive groups using linking agents, allowing for the covalent attachment of bioreceptors such as enzymes, antibodies, or nucleic acids [33]. Such as thiolated compounds to gold nanoparticles [32].

Selecting appropriate bioreceptors is crucial for minimizing interference from non-targeted molecules. Enzymes catalyse specific biochemical reactions, antibodies bind to specific antigens, and nucleic acids hybridize with complementary sequences, all of which enhance sensor specificity and accuracy by reducing false positives and ensuring detection of only the target molecules [34,35]. By considering the right combination, electrochemical biosensors can be improved to detect diabetes mellitus biomarkers more effectively and accurately.

### Comparison with literature

The development of biosensors has become increasingly important in the healthcare field, particularly with regard to diabetes mellitus. Laghlimi *et al*. [36] explored the application of electrochemical biosensors in various applications, particularly drug and metabolite detection, with emphasis on carbon-based electrodes such as screen-printed carbon electrodes (SPCE), glassy carbon electrodes (GCE), and carbon paste electrodes (CPE) as key components in constructing electrochemical biosensors and playing an important role in the detection of target molecules. In line with the review by Hovancová *et al*. [37], they discussed the use of carbon electrodes for glucose and insulin detection through electrochemical sensors, with sensor performance that can be improved through modification with nanomaterials.

In their study of recent advancements in the development of electrochemical biosensors for the diagnosis of diabetes mellitus, Pavla and Miroslav [38] emphasized new developments in the use of glucose, HbA1c, GHSA, and insulin biomarkers to monitor diabetes more effectively. Similarly, Yazdanpanah *et al*. [39] and Rescalli *et al.* [40] discussed the importance of biomarkers in the diagnosis and management of diabetes by highlighting the role of HbA1c and GHSA as biomarkers by examining the use of electrochemical biosensors to detect these biomarkers. On the other hand, Sabu *et al*. [41] discussed the development of biosensors for glucose and insulin monitoring in diabetes management by exploring the different types and detection mechanisms developed and providing prospects where device calibration and quality control should be performed to achieve good performance. A comparison of the related reviews is summarized in Table 1.

### Scope of the review

In this review, we will discuss the development of electrochemical biosensors to diagnose diabetes mellitus through their biomarkers using carbon-based electrodes that have been developed in the last decade. The layout of this review is designed as follows: *Introduction* gives the background and comparison with other literature to the scope of review of this article. Section *Diabetes mellitus biomarkers* discuss DM biomarkers that have a strong relationship with diabetes, such as glucose, HbA1c, GHSA, and insulin, as well as novel biomarker. Section *Electrochemical biosensors* discuss the electrochemical biosensor method in general and its potential for application in clinical applications. Section *Carbon-based electrode* provides insights into the selection of carbon-based electrodes for electrochemical biosensor development. Section *Electrochemical biosensor for diabetes biomarkers based on carbon-based electrode* presents electrochemical biosensors that have been developed for the detection of diabetes biomarkers based on widely used carbon-based electrodes (*i.e.* SPCE, GCE, CPE, BDD, graphene electrode, and graphite electrode). Operational principles, electrode modification strategies to improve sensitivity and specificity, and biosensor performance are discussed. Section *Open research issues* identifies opportunities and areas for further research. Lastly, *Conclusion* concludes the review with the summaries of the previous sections.

## Diabetes mellitus biomarker

Diabetes mellitus (DM) is a global epidemic disease, currently affecting around 537 million people in 2021, and is expected to continue to increase to around 783 million by 2045 [3]. Various factors can lead to the development of prediabetes, such as genetic abnormalities, glucotoxicity, insulin secretion abnormalities, impaired incretin release, amylin accumulation, lipotoxicity, oxidative stress, inflammation, and decreased β-pancreatic cell mass [42].

In general, DM can be classified into two main types: (i) insulin-dependent diabetes mellitus (IDDM) or type 1 DM, and (ii) non-insulin-dependent diabetes mellitus (NIDDM) or type 2 DM. Type 1 diabetes is primarily caused by the autoimmune destruction of insulin-producing beta cells in the pancreas, leading to insulin deficiency. Additionally, rare mutations can produce abnormal insulin molecules, and receptor abnormalities can impair insulin binding and glucose uptake, further influencing the disease [19,43]. Meanwhile, type 2 DM can arise from a complex interplay of factors, including insulin resistance, dysfunction of beta cells, chronic inflammation, adipocyte hormones, and genetic predisposition [44].

According to the American Diabetes Association (ADA) (2014) and World Health Organization (WHO) (2016), diabetes and prediabetes can be diagnosed by measuring fasting plasma glucose (FPG) and oral glucose tolerance test (OGTT) or by HbA1c concentration. On the other hand, several studies are currently looking at the performance of GHSA, insulin, and other biomarkers associated with type 2 diabetes mellitus and its complications. The ability to determine blood glucose over different time intervals and various influencing effects provides access to early diagnosis for more accurate diabetes prevention and treatment [45]. Each currently used diabetes mellitus biomarker diagnostics has its advantages and disadvantages, summarized in Table 2.

### Blood glucose

The carbohydrate monomer, blood glucose, is a crucial energy source for the body, typically stored as glycogen in the liver and skeletal muscles after assimilation. Through metabolic processes like glycolysis, glucose fuels various physiological functions. However, individuals with diabetes mellitus (DM) experience elevated blood sugar levels due to insulin dysfunction or resistance, impairing glucose uptake by cells [19]. Consequently, high blood glucose levels serve as a key biomarker for diabetes mellitus, contrasting with the normal range recommended by WHO (2016) and ADA (2020), which typically falls between 70-100 mg/dL during fasting and remains below 140 mg/dL two hours postprandial in healthy individuals. In DM, blood glucose levels exceed these thresholds, highlighting the significance of glycemic control in managing the condition.

Various diagnostic tests for diabetes rely on measuring blood glucose concentration, a fundamental and primary diagnostic procedure. Proper management of blood glucose levels is essential for preventing complications associated with diabetes mellitus [41]. Effective monitoring of glucose levels is crucial to diabetes management. In the past few decades, glucose biosensors have become an excellent tool to monitor glucose levels in real-time. Glucose biosensors have undergone several generations of development over time, in general, glucose biosensors can be divided into three generations [46]. The first-generation glucose biosensors use the enzyme glucose oxidase (GOx) to catalyse the oxidation reaction of glucose into gluconic acid and hydrogen peroxide. This reaction is then converted into an electric current by a transducer element [47]. This first-generation biosensor has been the cornerstone for the development of glucose biosensors. The second generation involved the use of electron mediators, such as ferrocene, to enhance electron transfer between the enzyme and the electrode, thereby improving the sensitivity and response of the biosensor [48]. The third generation introduced the use of nanomaterials, such as carbon nanotubes or metal nanoparticles, to improve electron transfer efficiency. This generation seeks to eliminate the use of artificial mediators and even enzymes themselves [49].

Glucose levels, which reflect the blood sugar balance, are a very important reflection in the effort to manage diabetes. The test results obtained help adjust therapy and provide early insight into the risk of diabetes. However, fluctuations in glucose levels can occur due to external factors such as changes in lifestyle and diet. Despite this, blood glucose monitoring remains crucial.

### Glycated hemoglobin (HbA1c)

Hemoglobin is a protein in red blood cells that acts as a transporter of oxygen from the lungs to body tissues and facilitates its return from tissues to the lungs. Hemoglobin (Hb) has a high affinity with oxygen due to its iron content. Each Hb molecule consists of four heme groups surrounding globin groups, forming a tetrahedral structure. Heme consists of iron ions in the centre of the organic compound porphyrin ring [50].

In normal adult humans, hemoglobin generally consists of 97 % adult hemoglobin (HbA) consists of two types of polypeptide chains, (α and β), hemoglobin A2 (HbA_2_) which contributes about 2.5 % with α and δ polypeptide chains as constituents; then about 0.5 % fetal hemoglobin (HbF), which is the main Hb in the fetus, is composed of α and γ polypeptide chains [51,52]. Hemoglobin A has a molecular weight of 64,458 Daltons [50].

**Figure 1.**
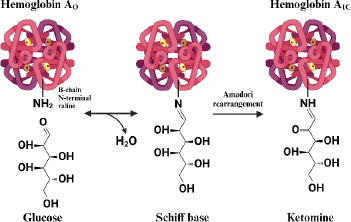
Illustration of HbA1c formation reaction (Redraw from [54] Copyright © 2023 by the authors).

Hemoglobin that undergoes a glycosylation process (HbA1c) is formed when the aldehyde group of glucose binds to the valine residue at the N-terminal of the β chain of the hemoglobin molecule to form an aldimide bond (Schiff base or labile HbA1c), this reaction is reversible then at a later stage an Amadori rearrangement occurs which produces an irreversible and more stable ketoamine [53]. The HbA1c formation reaction is shown in Figure 1.

Normally, glycolization reactions in adult hemoglobin (HbA) occur in 6 % of hemoglobin and 94 % of non-glycosylated hemoglobin (Hb). The 6 % of glycosylated HbA is also called HbA1, consisting of HbA1a and HbA1b, which are the minor components (1 %) and the major component, HbA1c (5 %) [55]. HbA1c has a stable hexose, glucose covalently bound to a valine residue at the NH_2_-terminal [56]. While HbA1a has fructose-1,6-diphosphate or glucose-6-phosphate, HbA1b has pyruvic acid. Each binds to a valine residue on the NH_2_-terminal of the β-chain [57]. Hemoglobin glycation reactions can also occur at sites other than the β-chain end, such as at the NH_2_-terminal valine residue of the α-chain as well as lysine residues at the α-chain or β-chain end [58].

The HbA1c level serves as a reliable indicator of the average plasma glucose level over two to three months, with a target of 6.5 % or less to reduce the risk of complications in diabetes mellitus [52]. According to the American Diabetes Association classification, HbA1c levels between 4 to 5.6 % are considered normal, 5.7 to 6.4 % indicate prediabetes, and levels above 6.4 % are indicative of diabetes [2]. Maintaining normal HbA1c levels is crucial for reducing the risk of microvascular complications and heart attacks in diabetic patients [59]. However, blood glucose monitoring using HbA1c still has limitations, which is inaccurate for patients with disorders that affect hemoglobin conditions such as hemoglobinopathy, iron deficiency, anemia, and other chronic kidney diseases and cannot reflect postprandial glycemia [60].

### Glycated human serum albumin (HbA1c)

One of the abundant proteins in the blood, albumin, has various functions, such as regulating osmotic pressure, playing a role in transportation binding, and having antioxidant properties [61]. Albumin can be a biomarker for blood sugar control in the form of glycated albumin (GHSA). Of the total blood protein, about 50% is human serum albumin (HSA) with a concentration of 35-50 g/L. This protein has a molecular weight of 67 kDa [62].

The formation of GHSA is directly related to hyperglycemia, the life span of albumin is about 2-3 weeks, making GHSA a biomarker of medium-term blood glucose control [63]. The formation of glycated albumin is reported to be 4.5 times faster glycated than hemoglobin [64]. Through non-enzymatic Maillard reactions such as HbA1c (Figure 1), albumin undergoes binding with glucose spontaneously on the amine groups of several residues such as arginine, lysine, and cysteine to form reversible Schiff base intermediate products, which then form ketoamine products through amadori rearrangement [63].

Early-stage glycation products, which undergo subsequent modifications such as oxidation, polymerization, cleavage, or rearrangement, are commonly referred to as advanced glycation end products (AGEs). The formation of AGEs from albumin is directly linked to the onset and progression of diabetic complications. Therefore, measuring glycated albumin not only provides information on blood glucose values but also indicates the progression of diabetes [65].

Diagnosis of diabetes becomes more reliable with the GHSA biomarker than HbA1c in patients who have kidney failure, anemia, or blood transfusion. GHSA measurement is based on the ratio of GHSA to total albumin [5]. There is no definite prediabetes cut-off level for glycated albumin, but one study used a level of ≥13.35 %, corresponding to an HbA1c level of 5.7 % (39 mmol/mol) to detect prediabetes. Meanwhile, the cut-off value for diabetes is ≥15.5 %. The combination of GHSA and HbA1c measurements for the diagnosis of prediabetes and diabetes can increase sensitivity compared to only HbA1c, besides that, GHSA can be an alternative biomarker in clinical conditions when HbA1c is inaccurate [66]. A limitation of glycated albumin is that it may lose its accuracy as a biomarker when there is a disturbance in albumin. In obese patients, glycated albumin levels can be lower due to higher albumin catabolism and low albumin production rates due to the effects of obesity-related inflammation [67].

### Insulin

**Figure 2.**
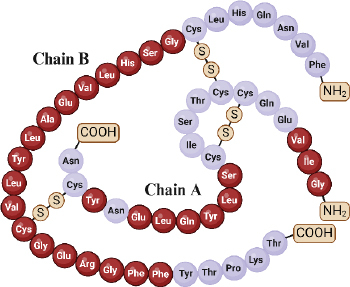
The structure of insulin with the active part of insulin is in red (Redraw [70] from Copyright © 2023 by the authors).

Insulin, an essential hormone in glucose metabolism, consists of two chains totalling 51 amino acid residues, with 21 in chain A and 30 in chain B. These chains are linked via disulfide bonds from the N-helix in chain A to the β-centre and C-terminus connecting chain A to the centre of chain B [68,69]. Insulin molecular weight is 5.8 kDa and has an isoelectric point at pH 5.5 [19]. The structural and functional integrity of insulin relies on specific amino acid residues in three regions of the A chain (positions 1-3, 12-17, and 19) and within the B chain (positions 8-25) (Figure 2) [70].

Insulin is produced within the pancreatic beta cells. Initially, it emerges as a signal peptide, undergoing synthesis into preproinsulin within ribosomes. Preproinsulin comprises the A and B chains alongside two additional domains—the signal domain and the C-peptide. The signal domain is eliminated in preproinsulin within the endoplasmic reticulum, transforming it into proinsulin. Proinsulin is then transported from the endoplasmic reticulum to the Golgi apparatus, where zinc and calcine are added, forming proinsulin hexamers. Outside the Golgi apparatus, enzymes cleave this proinsulin hexamer into insulin and C-peptide. Insulin is stored within B cell granules and secreted into the bloodstream when needed in response to elevated blood glucose levels [71,72].

Insufficient insulin secretion plays a pivotal role in the onset of diabetes. The normal fasting blood level of insulin is 25 mIU/L (0.86 ng/mL or 150 pM) [73]. Pre-diabetes occurs from the coexistence of insulin resistance and beta-cell dysfunction, which manifests before the onset of elevated blood glucose levels and eventually leads to diabetic complications [73]. Therefore, the detection of insulin levels is crucial in clinical diagnosis for the surveillance of pre-diabetes and diabetes, as well as the prevention of its complications.

### Novel biomarker

Nowadays, alongside traditional biomarkers, there's a growing focus on exploring novel ones. Numerous studies on DM biomarkers have highlighted the importance of novel biomarkers to gain a more comprehensive understanding of the DM condition. These novel biomarkers are crucial as they provide deeper insights into the complexity of factors influencing DM, thus enabling a more personalized approach to patient management. By leveraging these new biomarkers, therapies can be more precisely targeted, opening up opportunities for more effective DM control and prevention of associated complications.

**Figure 3.**
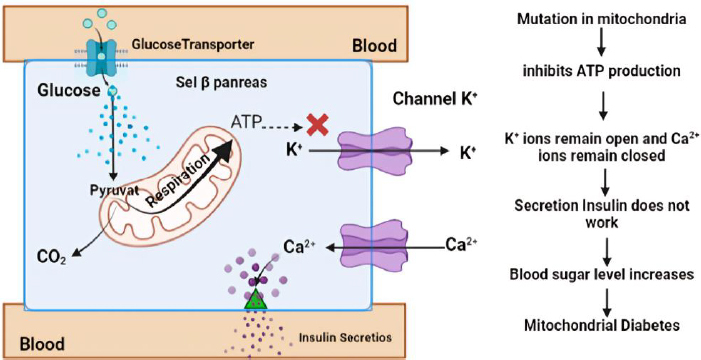
Mechanism of insulin secretion disruption in mitochondrial diabetes due to mtDNA mutations and ATP deficiency (Reprinted from [19] Copyright © 2023 by the authors).

One particular form of type 2 diabetes is mitochondrial diabetes, which accounts for 0.5 to 3 % of the overall diabetic population [74]. Mitochondrial diabetes is a condition caused by a genetic abnormality due to mutations in the mitochondrial DNA (mtDNA) gene that codes for a protein involved in the respiratory chain. High glycemia levels also increase the concentration of adenosine triphosphate (ATP) in pancreatic β-cells. This leads to the closure of K^+^ channels and causes membrane depolarization. The depolarization triggers the opening of Ca^2+^ channels. A sufficiently high level of Ca^2+^ in the cytosol activates insulin release through exocytosis (Figure 3). In patients with DM, ATP deficiency will inhibit insulin secretion and cause hyperglycemia [75]. Therefore, ATP can be used as a biomarker of DM, providing information on hyperglycemia occurring due to impaired insulin secretion caused by genetic disorders. Patients with this type of diabetes should avoid general diabetes medications like metformin, which inhibits mitochondrial respiration [76].

In understanding complications, sorbitol can be used as a biomarker where this sugar alcohol is formed through a reduction reaction by the enzyme aldose reductase. This enzyme is particularly active when glucose concentrations are high, as indicated by its high *K*_m_ value. Accumulation of sorbitol occurs predominantly in various bodily tissues, notably in the eyes, nerves, and kidneys. Due to its limited ability to escape these tissues, sorbitol can lead to the development of diabetic retinopathy, neuropathy, and nephropathy [77].

Another form of glucose, 1,5-anhydroglucitol (1,5-AG), is suggested as a marker for postprandial hyperglycemia, with its serum levels decreasing as serum glucose rises above the renal threshold for glucose [45]. Low concentrations of 1,5-AG in diabetes are indicative of hyperglycemic excursions over the prior 1-2 weeks. Furthermore, 1,5-AG is recognized as a biomarker for short-term glycemic control. Studies have indicated that 1,5-AG may serve as a valuable biomarker for prognosis related to microvascular outcomes in diabetes. Additionally, 1,5-AG has been proposed as a biomarker closely associated with decreasing functional β-cell mass before the onset of diabetes [78]. Research has explored the clinical advantages of combining serum 1,5-AG with fasting plasma glucose to identify diabetes in populations with hypertension, demonstrating its potential diagnostic value [79].

In individuals with type 2 diabetes, CRP (C-reactive protein) levels tend to be higher due to inflammation in certain tissues associated with insulin resistance and other complications of diabetes, such as the risk of cardiovascular disease, a serious complication among people with diabetes. Moreover, CRP has been associated with diabetic retinopathy, suggesting its potential role in monitoring diabetes-related complications [5].

Apart from insulin, other hormones, such as adiponectin, have been implicated in diabetes, particularly type 2 DM. Adipose tissue produces adiponectin in relatively small quantities, limiting its effectiveness in improving insulin sensitivity. This can contribute to insulin resistance, the main characteristic of type 2 diabetes, and adiponectin deficiency, which has been reported to be involved in gestational DM [80]. MicroRNAs have gained attention as biomarkers for diabetes, with specific microRNAs showing promise in predicting disease onset and progression. Studies have highlighted the role of microRNAs in the pathogenesis of chronic diseases, including diabetes, and their potential as diagnostic tools [81].

## Electrochemical biosensors

Generally, chemical sensors comprise two crucial functional components: a receptor or recognizer of chemical molecules and a transducer or mechanism that translates chemical reactions into a measurable signal using instrumentation [87]. On the other hand, biosensors are devices integrating biological elements with transducers to identify and quantify biochemical targets present in a sample. Of the various types of transducers, *i.e*., optical, piezoelectric, and thermal, used for biosensors, electrochemical transducers provide a simple yet efficient detection platform due to their ease in fabrication and integration of electrochemical cells. With this capability, electrochemical biosensors can provide fast response, high selectivity and sensitivity [88].

**Figure 4.**
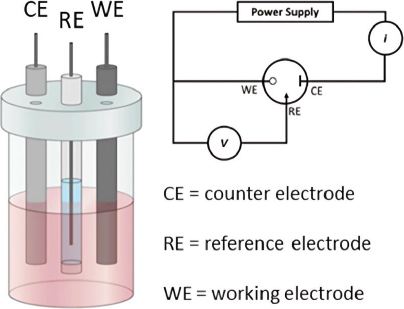
Electrochemical three-electrodes cell and its relative cell circuit (Reprinted from [92] Copyright © 2022 by the authors).

In electrochemical analysis, an electrochemical cell typically consists of two electrodes submerged in an electrolyte solution. There are two main types of electrochemical cells: galvanic (voltaic) cells and electrolytic cells. However, in electrochemical sensor or biosensor applications, a three-electrode configuration is often used [89] (Figure 4). This configuration includes the working electrode, which acts as the transducing element where the redox reaction occurs. The potential of the working electrode depends on the concentration of the analyte being measured. The reference electrode maintains a constant potential, independent of the analyte concentration, serving as a comparator to measure the potential at the working electrode. Lastly, the counter electrode ensures the passage of all the current necessary to balance the current at the working electrode [90,91]. This setup allows for more accurate potential measurements and better control of the reactions occurring within the electrochemical cell, which is crucial in various analytical and industrial applications.

Electrochemical biosensors detect the current produced by reduction or oxidation reactions, which is directly proportional to the concentration of electroactive species present [93]. Techniques such as voltammetry, amperometry, and chronoamperometry are employed to investigate electrochemical behaviour [94]. These techniques generate data based on changes in current, potential, impedance, and conductivity, which are then analyzed to elucidate reaction mechanisms at the electrode surface and calculate specific reaction constants [95]

Understanding the reaction mechanisms at the electrode surface typically involves concepts such as diffusion, adsorption, and reversibility of the system. The reaction rate is controlled by mass transfer (diffusion) [96]. In diffusion-controlled processes, the peak current (*i*_p_) is proportional to the square root of the scan rate (*v*^1/2^) as described by the Randles-Ševčik equation (1) [97]:


(1)

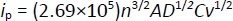



where *n* is the number of electrons transferred, *A* is the electrode area, *D* is the diffusion coefficient, *C* is the concentration, and *v* is the scan rate.

For adsorption-controlled processes, the peak current of a quasi-reversible system can be described by Equation (2) [98,99]:


(2)

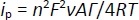



based on this equation, the surface concentration of the electroactive species (*Γ*) can be determined from the slope of the linear plot of *i* versus *ν*.

Laviron's theory is commonly applied to determine electron transfer rate constants for electron transfer between the electrode and surface-deposited layer, facilitated by parameters like standard rate constant (*k*_0_) and transfer coefficient (*α*). The theory relates the peak potential of an electrochemical reaction to the kinetic parameters of the system, such as the scan rate (*v*) [98,100]. The Laviron equation relates the peak potential of an electrochemical reaction to the kinetic parameters of the system, such as scan rate. It can be written by the Bard-Faulkner equation as follows for an irreversible system, Equation (3) [22]:


(3)

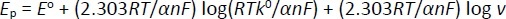



**Figure 5.**
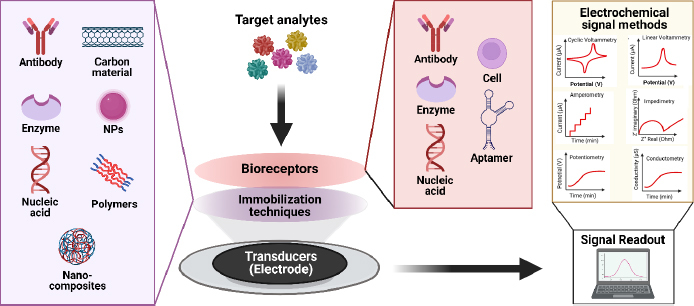
Schematic representation of an electrochemical biosensor.

The key element in electrochemical sensors lies in the presence of specific receptors capable of promoting the formation of complexes, enabling interactions to occur solely with the target and generate electrical signals, which are subsequently converted by electronic devices into an output. Biosensors utilize the specificity of biological recognition mechanisms by employing analyte recognition compounds such as enzymes, DNA probes, antibodies, aptamers, or proteins. Bioreceptors will form specific interactions with targets on the inter-surface, causing signal changes [101]. Subsequently, transducers capture signal changes from these interactions, converting them into continuous signals directly correlating with the number of molecules reacting or binding to the sensor surface. These signals can then be linked to a reading device for further analysis [102]. The concept of an electrochemical biosensor is shown in the schematic in Figure 5.

Electrochemical biosensor methods hold great promise for clinical applications due to their simple instrumentation, user-friendly operation, rapid measurement time, portability, and relatively lower cost. Currently, there is a significant focus on developing instruments for detecting biomarkers of diabetes mellitus, aiming to achieve the lowest possible detection limits while requiring only small sample volumes [103]. This focus on clinical applications underscores the importance of understanding and optimizing the electrochemical properties and reaction mechanisms at the electrode surface, as detailed through the aforementioned equations and techniques. By refining these methods, electrochemical biosensors can provide highly sensitive, accurate, and rapid diagnostic tools essential for effective disease management and monitoring [104].

## Carbon-based electrode

Carbon-based electrodes are commonly used in various electrochemical applications. Its main strengths include inert properties, chemical stability, and good compatibility with chemical and biological compounds. Moreover, good conductivity, affordable cost, and the ability to be functionalized in various ways to meet different application needs [100]. The inert nature of carbon makes it unreactive with various chemicals or harsh environments. As a result, it can be used under extreme conditions or for extended periods without undergoing significant degradation [105]. This makes carbon-based electrodes suitable for applications where high chemical stability is required, such as in environmental or pharmaceutical analysis. In addition, the biocompatibility of carbon-based electrodes makes them acceptable to the human body and can be used in medical equipment without causing any unwanted biological reactions [24,106].

Carbon-based electrodes offer several advantages over metal-based electrodes, such as gold and platinum. The production cost of carbon-based electrodes is relatively low compared to noble metal-based electrodes while maintaining good conductivity. Types of carbon-based electrodes, such as glassy carbon and diamond electrodes, match the conductivity of noble metals and offer a wider potential range, enabling a diverse array of electrochemical reactions [107-109]. Moreover, carbon electrodes demonstrate good biocompatibility, causing minimal toxic reactions in biological tissues and showing reduced susceptibility to biofouling, which can impede electrode functionality. In contrast, while metal materials also have good biocompatibility, they can cause allergic reactions or irritation in some individuals and are more susceptible to biofouling [110]. Carbon-based electrodes are ideal in healthcare applications due to their high biocompatibility, ease of sterilization, and lower risk of contamination, making them suitable for single-use applications. Meanwhile, noble metal electrodes are often more expensive and less practical for disposable applications due to their higher cost and limited availability [111,112]. For a detailed comparison between carbon-based electrodes and other electrode materials, see Table 3.

In addition, carbon-based electrodes can also be functionalized in various ways to suit different application needs. They can be functionalized by electrochemical film deposition methods, the use of carbon nanomaterials, or other surface modifications to improve electrode functionality [26]. With these advantages, carbon-based electrodes continue to be a highly desirable material in the development of sensors, biosensors, and various other electrochemical applications.

## Electrochemical biosensor for diabetes biomarkers based on carbon-based electrode

In electrochemical applications, the electrode plays a crucial role as the site where reactions occur, particularly in electrochemical biosensors, where it serves as the interface between biological and electronic systems [112]. Carbon electrodes are commonly preferred for such applications due to several compelling reasons mentioned in the previous section. Furthermore, their capacity for chemical modification enables customization according to specific application requirements, including enhancing the sensitivity, specificity, or lifetime of the biosensor [24,25]. In this section, we explore the development of electrochemical biosensors for the detection of diabetes biomarkers based on widely used carbon-based electrodes, which are GCE and SPCE, and also other electrodes that are less commonly used in electrochemical biosensors for the detection of DM biomarkers but have been reported. It delves into operational principles and electrode modification strategies to enhance biosensor performance.

### Glassy carbon electrode

Glassy carbon electrodes (GCE) have been developed for electrochemical sensors since around the 1960s [95]. This electrode is one of the most widely used and applied electrode types in electroanalysis due to its advantages of electrochemical inertness over a wide potential window, chemical stability, ease of surface modification, and robustness [107]. Additionally, glassy carbon (GC) has some interesting physicochemical properties, including minimal thermal expansion, excellent biocompatibility, very low gas and liquid permeability [121], excellent thermal (0.7 to 4 W/m K) and electrical (10 to 10000 S/m) conductivity [122].

Synthesized via a bottom-up approach involving the pyrolysis of specialized polymers at extreme temperatures. This carbonization process produces a carbon-based material that does not produce graphite. The high density and low porosity of the GC structure provide strong and durable mechanical properties. Its three-dimensional graphene structure also offers high thermal and electrical conductivity, making GC a common choice in electrochemical sensor applications. Furthermore, the amorphous and non-porous glass-like structure of GC enhances its resistance to corrosion and chemical reactions [123,124].

In recent advancements, Li *et al*. [125] have developed glucose biosensors using modified GCE by incorporating a blend of glucose oxidase (GOx) with hydroxy fullerene (HF), bovine serum albumin (BSA), and multi-walled carbon nanotubes (MWCNT) shielded by glutaraldehyde (GA)/Nafion (NF) composite membrane to prevent enzyme damage. The HF-GOx complex can enhance the electron conductivity and catalysis of glucose oxidation reaction, with BSA improving the low biocompatibility and balancing the hydrophobicity of MWCNTs-HFs. Modification of GCE with carbon in the form of MWCNTs enhances electrical conductivity and provides a large specific surface area, facilitating enhanced interaction with GOx [126]. This glucose biosensor exhibits remarkable sensitivity (167 μA/mM cm^2^) and a low detection limit (17 μM) with a resulting Michaelis-Menten constant of 119 μM. Validation tests on human blood plasma samples confirm its efficacy in detecting glucose concentrations with satisfactory recovery rates [125].

Karaşallı *et al*. [127] developed an innovative electrochemical immunosensor for label-free detection of HbA1c using reduced graphene oxide (ERGO). HbA1c antibody was immobilized on ERGO/GCE via physical adsorption with van der Waals interactions and electrostatic forces. Physical adsorption allows the antibody greater flexibility and mobility to move and adapt to the structure of the HbA1c antigen. This immunosensor has a detection range between 1 to 25 %, with high sensitivity in detecting HbA1c in human serum samples.

**Figure 6.**
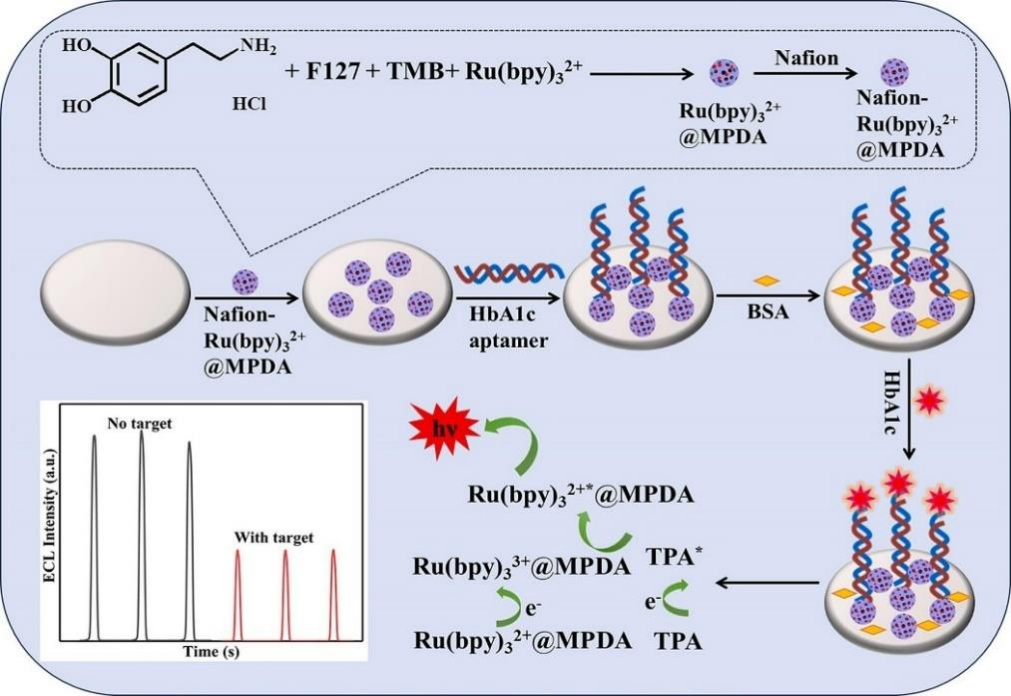
Schematic of ECL immunosensor based on Ru(bpy)_3_^2+^ @MPDA to detect HbA1c (Reprinted from [129] Copyright © 2020 Elsevier B.V.).

The electrochemiluminescence (ECL) method in biosensors works by involving a chemiluminescence process initiated by electrochemical methods. This process involves the use of electrodes to initiate a chemical reaction that results in photon emission. The ECL method offers sensitivity, a wide linear range, and excellent selectivity [128]. Zhang *et al.* [129] developed an ECL biosensor for HbA1c detection using Ru(bpy)_3_^2+^ modified GCE as a chemiluminescence reagent encapsulated in mesoporous polydopamine (MPDA) (Figure 6), forming a substrate capable of conjugating HbA1c aptamer through amidation reaction. This aptamer-based biosensor showed a wide linear range from 0.1 to 18.5 % with a low limit of detection (LOD) of 0.015 %.

Nanozymes possessing enzyme-like properties have been applied to GHSA biosensors using GCE electrodes by Li *et al*. [130]. In their study, GCE was modified with copper oxide (Cu_2_O) modified reduced graphene oxide (rGO) nanocomposite, functioning as a nanozyme akin to GOx.

In the presence of the target, GHSA will be captured by methylene blue-labeled DNA tripods (MB-tDNA), resulting in a decrease in the MB-tDNA reduction current and an increase in the oxidation current due to enhanced exposure of the catalytic surface to nanozymes. Measurement of GHSA from serum samples was monitored from the ratio of glucose oxidation and methylene blue reduction currents (iGlu/iMB) using differential pulse voltammetry (DPV), the linear range and limit of detection offered from this biosensor are 0.02-1500 μg/mL and LOD 0.007 μg/mL, respectively.

**Figure 7.**
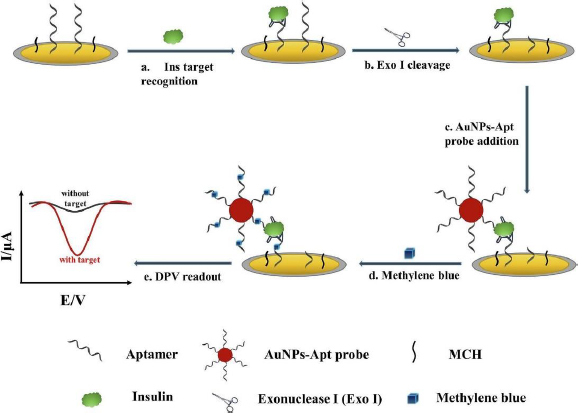
Construction of a 'sandwich' structure in a biosensor for insulin detection using aptamer, exonuclease I, and AuNP-Apt probe (Reprinted from [131] Copyright © 2021 Elsevier B.V.).

Liu *et al*. [131] developed an electrochemical aptasensor for insulin detection, employing a modified 'sandwich' structure system on the electrode. In this context, 'sandwich' refers to the complex structure formed between insulin with one side bound to the aptamer deposited on AuNP/GCE, and the other side bound to gold nanoparticles-aptamer (AuNPs-Apt) (Figure 7). This 'sandwich' structure enables amplification of the electrochemical signal from methylene blue (MB) intercalated into the guanine base of the aptamer. Employing this strategy resulted in high insulin sensitivity, evidenced by the remarkably low detection limit of 9.8 fM and a wide linear range from 0.1 pM to 1.0 μM.

From the limited number of studies, a few researchers have developed DM detection methods using novel biomarkers with electrochemical biosensors that employ carbon-based electrodes. Among these studies, some have reported using GCE. ATP is a novel biomarker for DM, specifically mitochondrial diabetes. Maksum *et al*. [132] explained that mtDNA mutations disrupt protein respiration function and decreased ATP production. This insufficiency or absence of ATP affects the insulin secretion process.

**Figure 8.**
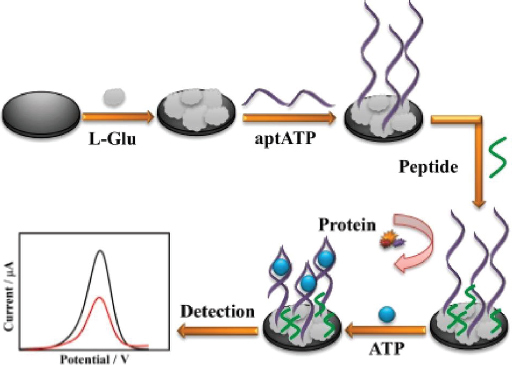
Schematic illustrating the fabrication process of the ATP aptasensor (Reprinted from [133] Copyright © 2021 Elsevier B.V.).

Meng *et al*. [133] developed a highly sensitive and anti-fouling electrochemical aptasensor for detecting ATP. The electrode was modified by electrodeposition of poly(glutamic acid) (p-L-Glu), followed by the immobilization of aptamer and peptide (Figure 8). The p-L-Glu serves multiple purposes: enhancing electrode conductivity through its conductive groups, providing a matrix for ATP aptamer immobilization, and increasing the hydrophilicity of the electrode interface to prevent non-target molecules (particularly hydrophobic ones) from interfering, thus improving anti-fouling ability. The aptasensor demonstrated excellent selectivity and sensitivity, with a linear detection range from 0.01 pM to 1.0 μM. This range encompasses the concentration of ATP typically found in the human body (nanomolar to micromolar).

C-peptide, a by-product of proinsulin cleavage, serves as a biomarker of DM. Unlike insulin, C-peptide is not influenced by external factors such as insulin injections. Therefore, detecting C-peptide can offer more precise information about pancreatic beta cell function. Using an electrochemical biosensor based on electrochemiluminescence, Wang *et al*. [134] used GCE-modified hairpin DNA probes containing dopamine that function to increase signal sensitivity. Dopamine reduces the ECL signal by inhibiting the Ru-PEI-ABEI complex, which is a strong signal source. Glucose detection relies on decreasing ECL signal with increasing C-peptide. This biosensor has a detection limit of 16.7 fg/mL.

The electrochemical biosensors utilizing GCE developed in the last decade for detecting DM biomarkers are summarized in Table 4.

### Screen-printed carbon electrode

In clinical settings, the utilization of disposable devices has become commonplace to uphold stringent hygiene protocols, prevent cross-contamination, maintain consistency of performance, and ensure safety for patients and medical staff. Screen-printed carbon electrode (SPCE) is widely used for disposable sensor fabrication. Its simplicity, ease of operation, portability, and mass production make SPCE a suitable candidate for clinical diagnostics. SPCE comprises three electrodes used in electroanalysis: a working electrode, reference electrode, and counter electrode constructed as a miniaturized electrochemical cell [111]. Any type of carbon is possible to be deposited by a screen-printing technique, commonly used carbons are carbon black and graphite. Other carbons have also been examined for use, such as carbon nanotubes, carbon nanofibers, and graphene [147].

Fabrication of screen-printed carbon electrodes (SPCE) relies on screen-printing technology, where carbon ink is deposited onto a thin, flat substrate using a layer-by-layer deposition technique to create the desired electrode pattern [148]. However, reproducibility is hindered by variability in the moulding process, including factors like molding pressure, humidity, or temperature. Differences in material batches and contamination during fabrication may also contribute to variations in electrode performance and electrochemical properties [149]. Nonetheless, the combined simplicity and miniaturization capabilities in these electrodes render them ideal for various electrochemical applications, including the detection of various biomarkers of diabetes mellitus.

Fiérrez *et al*. [150] developed an electrochemical biosensor for the enzymatic detection of glucose using GOx with SPCE modified with poly(azure A) (PAA) to immobilize GOx and enhanced electrodeposition of platinum nanoparticles (PtNP) on the electrode surface. Platinum catalyses the oxidation of H_2_O_2_ generated during glucose oxidation, contributing to the stability and reproducibility of the biosensor [151]. GOx coated on the SPCE surface will oxidize glucose by involving O_2_ and flavin adenine dinucleotide (FAD), producing gluconolactone and the intermediate product H_2_O_2_, which will release electrons and transferred to the electrode, generating a measurable electric current [141]. This biosensor exhibits an excellent sensitivity of 42.7 μA/mM cm^2^ with a low detection limit of 7.6 μM and a wide linear range [150].

Eissa *et al*. [27] developed an electrochemical aptasensor for HbA1c detection. The biosensor was fabricated by electrodeposition of gold nanoparticles (AuNPs) on SPCE. AuNPs serve to immobilize biomolecules and enhance the current response due to gold's high conductivity and electrocatalytic properties [152]. The thiolated aptamer interacts with AuNPs, forming a covalent bond. Aptamers as bioreceptors are single-stranded oligonucleotides with a specific affinity to target molecules due to their 3D structure [153]. This aptasensor was tested on diluted blood samples, the presence of HbA1c in the sample causes the oxidation current [Fe(CN)_6_]^4−/3−^ to be blocked. The detection limit value was shown to be very low at 0.2 ng/mL [154].

**Figure 9.**
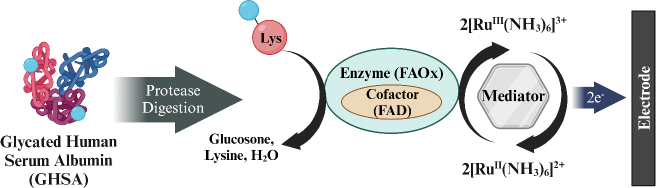
Principle of GHSA measurement via fructosyl lysine (ε-FK) oxidized by FAOx and Ru-complex (Redraw using © 2024 BioRender from [155] Copyright © 2016 Elsevier B.V).

Hatada *et al*. [155] developed a biosensor for detecting GHSA using fructosyl amino acid oxidase (FAOx) enzyme and hexaammineruthenium(III) chloride (2[Ru^III^ (NH_3_)_6_]^3+^) modified on SPCE. GHSA measurement involves a degradation reaction by proteases on GHSA to release ε-fructosyl lysine (ε-FK). The Ru complex is reduced simultaneously with the oxidation of ε-FK by FAOx, and the amount of reduced 2[Ru^III^ (NH_3_)_6_]^3+^ is measured using chronoamperometry (CA) to determine the concentration of GHSA in the blood sample (Figure 9). This biosensor has a wide detection range from 0 to 100 μg/mL with a detection limit of 0.1 μg/mL.

**Figure 10.**
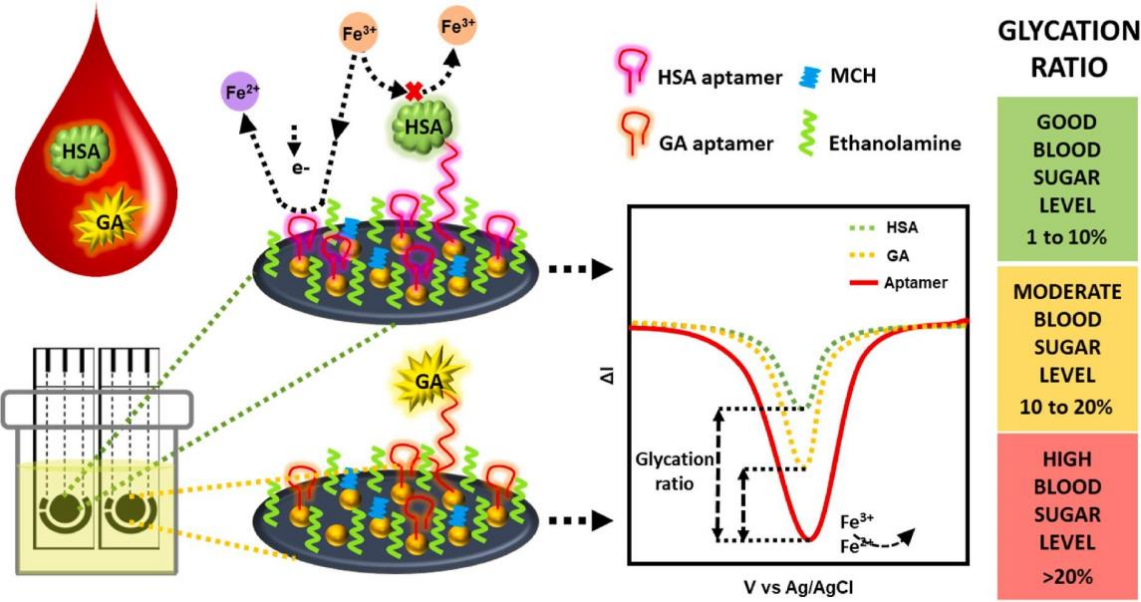
Schematic representation of aptasensor for GHSA detection by looking at the HSA/GHSA ratio using two aptamers that are selective for each of them (Reprinted from [156] Copyright © 2023 The Authors).

Recent advancements in GHSA biosensors have also been made by Dastidar *et al*. [156], utilizing thiolated aptamers bound to gold nanoislands (AuNI) to increase the detection sensitivity of biomolecules. The irregular structure and large surface area of AuNI provide more functionalization sites and interaction with the aptamer, then MCH and ethanolamine use for double blocking of the free sites (Figure 10). The immobilized aptamer reduces the redox current due to the electrostatic repulsion between the [Fe(CN)_6_]^4-/3-^ anion and the phosphate backbone of the negatively charged DNA aptamer [27]. This biosensor is capable of detecting GHSA and HSA simultaneously in biological samples with a clinically relevant concentration detection range of 1-40 mg/mL for GHSA and 20-60 mg/mL for HSA.

A screen-printed carbon electrode modified with ordered mesoporous carbon (OMC) and 1,3,6,8-pyrenetetrasulfonate (TPS) showed aptamer-based insulin detection with good sensitivity and selectivity. Modification of the electrode with OMC can increase the contact area between the electrode and the sample, while TPS facilitates the immobilization of aptamer on the electrode surface by binding the aptamer to TPS sulfonate through the cross-reaction of aryl sulfonate chloride. The detection mechanism of insulin relies on the change of MB signal. Following the interaction of insulin with the aptamer probe on the SPCE surface, MB desorbs from the electrode surface, leading to a decrease in the signal detected through DPV. Insulin measurements can be performed on human serum samples, albeit with 10,000-fold dilution. The detection range offered by this insulin biosensor is very wide, from 1.0 fM to 10.0 pM, with a very low detection limit of 0.18 fM [29].

Besides GCE, the SPCE electrode has also been prominently utilized in developing electrochemical biosensors for detecting novel biomarkers such as ATP, CRP, microRNA, and 1,5-AG. This indicates that SPCE is generally used among the various electrodes available. Recently, Mulyani *et al*. [152] developed an electrochemical biosensor that selectively detects ATP using an aptamer from Kashefi-Kheyrabadi's research [157]. The thiolated aptamer was immobilized on SPCE through bonding between thiol groups and gold groups deposited by the drop-casting method. The voltammograms from characterization using DPV showed selective results between ATP and UTP, CTP, and GTP. Furthermore, Rustaman *et al.* [158] employed an in silico method to demonstrate that this aptamer also exhibits selectivity towards ADP and AMP. The developed biosensor has a limit of detection and limit of quantification of 7.43 and 24.78 μM, respectively, with a linear range of 0.1 to 100 μM [152].

**Figure 11.**
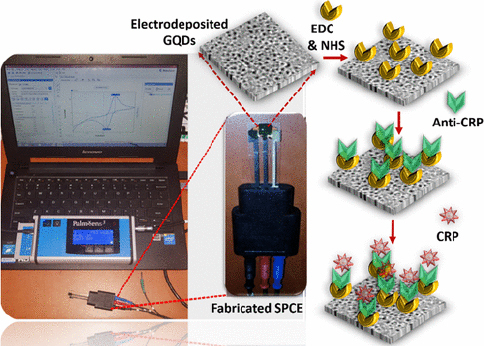
Illustration of an antibody-based electrochemical biosensor for detecting CRP using SPCE/GQDs (Reprinted from [159] Copyright © 2021 The Authors. Licensed under CC-BY-NC-ND 4.0)

CRP is an inflammatory biomarker that can indicate inflammation in the body associated with various conditions, including type 2 diabetes. Detection of CRP can help in monitoring the body's inflammatory state and response to treatment or lifestyle changes in individuals with diabetes to reduce the risk of serious complications such as heart disease and stroke. Lakshmanakumar *et al*. [159] have fabricated SPCE with graphene quantum dots (GQD) to improve the sensitivity of CRP detection compared to using carbon nanotubes and gold nanoparticles, which are reported to be less sensitive for label-free CRP detection. This immunoassay-based biosensor utilizes EDC:NHS to form a stable amide bond between the carboxyl terminal group of GQDs and the amine group of anti-CRP (Figure 11). Electrochemical detection of CRP was performed by amperometry and DPV in artificial blood solution. Results showed that the biosensor has a high sensitivity of 2.45 μA/ng mL cm^2^ with a linear range of 0.5-10 ng/mL, and a detection limit of 0.036 ng/mL.

**Figure 12.**
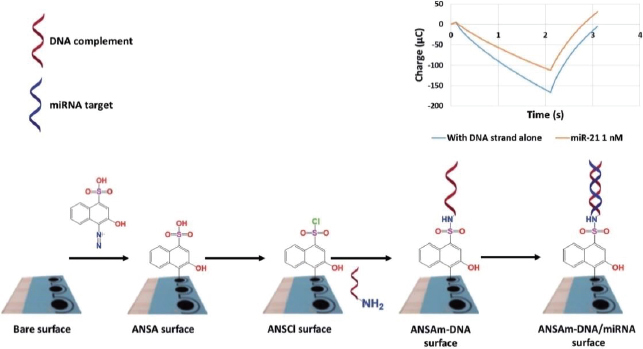
Illustration of fabricating an miRNA biosensor from SPCEs, involving hybridization with a target miRNA (Reproduced from [160] with permission from the Royal Society of Chemistry)

MicroRNAs are involved in the regulation of glucose metabolism, inflammation, oxidative stress, and diabetic nephropathy complications. These small RNA molecules have been detected by Daniel *et al*. [160] using DNA strand immobilized on diazo sulphonamide modified SPCE from 4-amino-3-hydroxy-1-napthalene sulfonic acid (ANSA) solution. ANSA will turn into sulfonyl chloride (ANSCl) to form sulphonamide bonds with oligonucleotides (Figure 12). The samples used in this biosensor are urine samples from diabetic kidney disease (DKD) patients. This study was able to detect both miR-192 (associated with DKD) and miR-21 (associated with oxidative stress) with a detection limit of 17 fM.

1,5-AG is a deoxyglucose form that can reflect postprandial glucose levels dynamically. The normal concentration of 1,5-AG ranges from 12-40 μg/mL, but may decrease in patients with DM. Recently, Li *et al*. [161] developed an electrochemical biosensor using SPCE electrode modified with Persimmon-Tannin-Reduced Graphene Oxide-PtPd (PT-rGO-PtPd) nanocomposite to detect 1,5-AG. The 1,5-AG detection mechanism involves the enzyme pyranose oxidase (PROD) bound to the modified electrode to catalyse the oxidation of 1,5-AG to 1,5-anhydrofructose (1,5-AF) and H_2_O_2_. The measurement results by DPV showed a detection range of 0.1-2.0 mg/mL and a detection limit of 30 μg/mL. Table 5 summarizes the electrochemical biosensors developed in the past decade that employ SPCE for detecting DM biomarkers.

## Others carbon-based electrodes

### Carbon paste electrode

Carbon paste electrode (CPE) is one of the promising electrodes in electrochemical sensors and can be widely applied due to its advantages of chemical inertness, low background current (compared to solid graphite or rare-earth metal electrodes), low ohmic resistance, environmentally friendly, non-toxic and adaptability to various detection applications. Moreover, the passivation problem encountered with CPEs can be resolved swiftly and easily renewing the electrode surface [26,173]. However, despite these benefits, CPE has certain limitations when used in electrochemical sensors, including lower reproducibility and sensitivity, as well as the requirement for higher overpotentials for electrocatalytic processes and slower electron transfer. Nevertheless, these challenges can be overcome through electrode modification [173]. The fabrication of CPE involves blending carbon powder with a binder like paraffin oil or silicon oil, with the resulting soft carbon paste being filled into an electrode holder or container and then solidified [174]. In biosensor development, CPEs can be chemically modified with specific target recognition materials to enhance target detection specificity [175].

Donmez *et al*. [176] studied the utilization of poly L-aspartic acid (PAA) modified CPE electrodes for glucose detection. The modification process involved immersing the electrode in a PBS solution containing poly L-aspartic acid, followed by cyclic voltammetry (CV) to polymerize PAA. The immobilization of GOx on PAA/CPE relied on the covalent binding of GOx to the carboxyl group of N-hydroxysuccinimide (NHS) and 1-ethyl-3-(3-dimethylaminopropyl) carbodiimide (EDC)-activated PAA. The amperometry use to measure glucose within a concentration range of 0.01-1.0 mM, demonstrating good selectivity by disregarding interfering species such as dopamine, ascorbic acid, and uric acid, with the time required to achieve 95 % steady-state current being less than 4 seconds.

An activated carbon-based CPE electrode was developed for glucose biosensor by Fatoni *et al*. [28] with a modified NiFe_2_O_4_ nanoparticle composite. The use of activated carbon aimed to enhance characteristics such as large surface area and high electrical conductivity. This modified CPE exhibited optimal performance with the inclusion of 8% NiFe-NPs, enhancing the conductivity and catalytic properties of the biosensor. Compared to the standard hospital method of detecting glucose in blood samples, this biosensor yielded values that were not significantly different, indicating precise results suitable for medical applications, with a linear response ranging from 2 to 10 mM.

### Boron-doped diamond

Boron-doped diamond (BDD) has gained attention in the development of electrochemical sensors. As a variant of carbon (sp^3^ hybridization) modified by doping boron atoms into the diamond structure, BDD is reportedly 'metal-like' as it offers high conductive properties, different from the insulating properties of diamond [109]. This unique characteristic, along with its high chemical stability, wide potential window, and corrosion resistance, positions BDD as a highly promising electrode material for biosensor applications [177]. The fabrication of BDD electrodes using the chemical vapor deposition (CVD) method enables precise control over their structure and composition. This process involves decomposing carbon- and boron-containing precursor gases on a hot substrate, creating a BDD layer with the desired conductive properties [109]. The BDD electrodes can be efficiently reused for various applications through surface treatment. This unique characteristic allows them to restore their surface to its original condition through treatment under extreme conditions, such as using extremely high acidity (*e.g*. aqua regia) and electrocleaning with very high potentials (reduction/oxidation). Unlike BDD electrodes, aggressive or complex treatment processes may potentially damage or alter the properties of other types of carbon electrodes [178].

In glucose detection applications, BDD electrodes can be employed directly or indirectly for oxidation through functionalization with specific enzymes. Their high sensitivity and long-term stability make BDD electrodes an ideal choice for developing reliable electrochemical biosensors to monitor glucose levels in diabetes [179]. Fachrurrazie *et al*. [180] successfully developed an enzymatic glucose biosensor with a BDD electrode modified to be nitrogen-terminated and coated with AuNPs. The formation of a bond between NH_2_ and AuNPs covalently resulted in a more stable immobilization of GOx on the electrode. The reduction peak on the GOx/AuNP/BDD electrode showed a linear response to glucose concentration with R^2^ of 0.99.

To create a sensitive and minimally interfered glucose detection method using a BDD electrode, Yoon *et al*. [181] recently combined a modified BDD electrode with H_2_O_2_/NH_4_OH and the electron mediator menadione. This approach utilized the oxidation reaction of glucose by FAD-GDH for the electrochemical-enzymatic (EN) method and DT-D enzyme with NAD-GDH for the electrochemical-enzymatic-enzymatic (ENN) method. The biosensor demonstrated a detection limit of about 20 and 3 μM in redox cycling of EN and ENN, respectively. Due to the slow redox reaction between menadione and interference species such as ascorbic acid, uric acid, and acetaminophen, this biosensor can selectively detect glucose with high sensitivity.

### Graphene

Graphene is a two-dimensional material with a single layer of carbon atoms arranged in a hexagonal structure. Graphene has unique properties such as high electrical conductivity (64 mS/cm), good mechanical strength (Young's Modulus, *E* = 1.0-1.02 TPa), good chemical stability, large surface area (2600 to 2630 m^2^/g), and high mobility of biomolecules (10,000-15,000 cm^2^/V), make it highly attractive for various applications, including biomedical sensors [182,183]. Recent advancements in graphene electrode fabrication include laser-induced graphene (LIG) and laser-scribed graphene (LSG) technologies. These methods utilize laser writing to transform carbon substrates into 3D graphene structures. By optimizing parameters like laser speed and power, these techniques can produce graphene electrodes with precise properties and large surface areas due to their porous structure. These characters enable good adhesion of target biomolecules and increase the sensitivity of biomarker detection [184,185].

**Figure 13.**
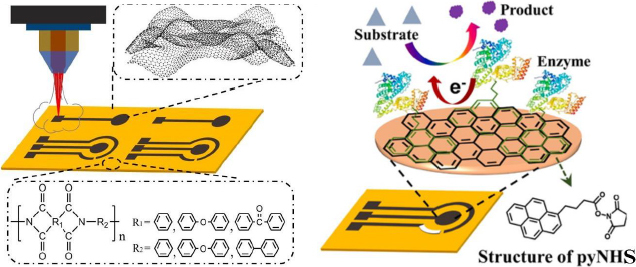
Illustration of laser-scribed graphene fabrication (left) and schematic of enzymatic glucose biosensor using pyNHS (right) (Reprinted from [186] Copyright © 2023 Elsevier B.V.).

Luo *et al*. [186] developed a biosensor focusing on enhancing the electrochemical performance of the LSG electrode for sensitive glucose detection. In this study, the LSG electrode was modified with the GOx enzyme using 1-pyrenebutyric acid N-hydroxysuccinimide ester (pyNHS) as a heterobifunctional linker attach GOx to the working electrode surface (Figure 13). Detection of H_2_O_2_ formed from the glucose oxidation reaction by GOx was performed by amperometry, resulting in a detectable concentration range of 0.04 to 4.0 mM with a sensitivity of 16.35 μA/mM cm^2^.

Besides LSG, the LIG electrode has also been used in enzymatic electrochemical biosensors for glucose detection. Liu *et al*. [187] developed a GOx/Fc/LIGE electrode that exhibited high sensitivity (11.3 μA/mM cm^2^), a wide linear range of detection (0-11 mM), and a low detection limit (0.04 μM). This electrode is flexible, capable of bending up to 60° without a significant change in conductivity and demonstrates good repeatability in detecting glucose in serum samples. For insulin detection, Liu *et al*. [131] employed LSGE to develop an electrochemical aptasensor, modifying the electrode surface to form a 'sandwich' structure (AuNP-Apt/insulin/ /aptamer) based on optimization results on GCE. The detection limit for LSGE in insulin detection is 22.7 fM. This biosensor was designed as a disposable electrode platform to reduce the risk of cross-contamination between different samples and showed good consistency (RSD = 1.80 %) in the modified results.

### Graphite

Graphite is a versatile crystalline carbon material widely used in various electrochemical biosensor applications. Similar to graphene, graphite possesses a layered structure. Various forms of graphite electrodes, including graphite fibre microelectrodes (GFE), graphite rods (GR), pencil graphite electrodes (PGE), and graphite sheets (GS), have been applied in biomarker detection. GFE, with its large surface area and high electrical conductivity, offers optimal sensitivity in the detection of target molecules in very small solutions [188]. On the other hand, graphite rods that are commonly used provide good mechanical stability and are easily modifiable to enhance the specificity of biomarker detection [189]. Then PGE, known for its affordability and accessibility, exhibits good sensitivity [190], while GS allows for intricate design possibilities and integration with advanced sensor technologies [191]. The advantages of graphite electrodes include high electrical conductivity, mechanical stability, and flexibility in design and application. The synthesis of graphite electrodes involves methods such as pressure-controlled deposition, CVD, the redox method (Hummer's method), and thermal decomposition of graphite [192], depending on the desired application. In biomarker detection, graphite electrodes have been successfully used in the diagnosis of diseases such as diabetes and neurological diseases [191,193].

**Figure 14.**
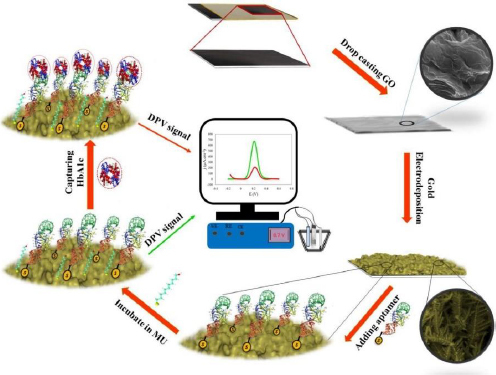
Aptasensor for HbA1c detection using GS as the substrate for aptamer adsorption (Reprinted from [191] Copyright © 2019 Elsevier B.V.).

Jaberi *et al*. [191] developed a GS-based electrochemical biosensor modified with reduced graphite-gold (rGO-Au) nanocomposite to detect glycated hemoglobin (HbA1c) levels in blood samples (Figure 14). The rGO-Au nanocomposite increases the surface area and electron transfer on the electrode surface, facilitating the binding of the thiolated DNA aptamer bioreceptor to form a self-assembly monolayer (SAM) with gold. The presence of HbA1c caused a decrease in current detected by DPV using the redox probe Fe(CN)_6_^3-/4-^. This label-free biosensor showed high sensitivity (269.2 mA/cm) and a wide linear range (1 nM to 13.83 mM). However, the HbA1c concentration in the blood sample needs to be diluted as it exceeds the linear range of the biosensor.

The sensitivity of the GR electrode-based glucose detection biosensor can be improved using dendritic gold nanostructures (DGN). Sakalauskiene *et al*. [189] studied the immobilization method of GOx by cross-link method using glutaraldehyde (GA). The surface of the DGN/GR electrode was converted into a carboxylic acid layer using 11-mercaptoundecanoic acid, which can form a SAM on top of DGN. Subsequently, GOx was covalently immobilized onto the modified SAM with GA as a cross-linking agent. GA helped enhance the repeatability of the current response and reduce the damage or detachment of DGNs from the electrode surface along with the enzyme under inappropriate experimental conditions. This study gave the highest Δ/_max_ of 384.20 ± 16.06 μA using GA-GOx-SAM/DGNs/GR electrode and linear dynamic range from 0.1 to 10 mM in serum samples.

Low levels of adinopectin have been linked to insulin resistance, obesity and cardiovascular disease. Adiponectin has an important role in regulating lipid and glucose metabolism and has anti-inflammatory and antioxidant properties. Adiponectin can improve insulin sensitivity, reduce inflammation in the blood vessel wall, and inhibit the proliferation of smooth muscle cells in blood vessels. Therefore, increasing adiponectin levels in the body may be a potential strategy to prevent and treat diabetes and its complications. One of the recent studies by Özcan and Sezgintürk [194] has proposed an innovative biosensor system to detect adiponectin in human serum. This biosensor uses a graphite paper (GP) electrode as the working electrode, with antiadiponectin as the bioreceptor of adiponectin. The system using the GP electrode produces sensitive detection with measurement methods using electrochemical impedance spectroscopy and CV. This biosensor is able to detect adiponectin levels at the picogram level with a wide detection range (0.05 to 25 pg/mL) and low detection limit (0.0033 pg/mL). Besides that, the electrode used shows the ability to be regenerated up to 18 times. A summary of electrochemical biosensors developed over the last decade using CPE, BDD, graphene electrodes, and graphite electrodes to detect DM biomarkers is presented in Table 6.

## Open research issues

In current clinical practice, the early diagnosis of diabetes often relies on blood glucose concentration due to its simplicity in measurement using a glucometer. However, this measurement does not always provide a fully accurate diagnosis of diabetes. Other highly correlated biomarkers such as HbA1c, GHSA, and insulin are now becoming important research subjects in the diagnosis and monitoring of diabetes. These biomarkers can provide more in-depth information about the duration of hyperglycemia, the risk of complications, and the insulin-related causes of diabetes, which are impaired insulin secretion or insulin production and insulin resistance.

Electrochemical biosensor methods offer a formidable alternative for disease diagnosis and monitoring. Compared to gold-standard methods like HPLC and ELISA for biomarkers, electrochemical biosensor methods excel in developing point-of-care diagnostics due to their simplicity, speed, portability, and reliability. Carbon-based electrodes are highly suitable for clinical applications due to their cost-effectiveness, making them potentially accessible to the general public. Additionally, these electrodes are inert and biocompatible. Their inert nature ensures that the resulting signal originates from a specific interaction with the target biomolecule, which is necessary for accurate diagnosis. However, carbon-based electrodes may lack sensitivity, improving sensitivity and electrocatalytic activity can be addressed by electrode modification strategies, such as metal nanoparticles or reduced/oxidized carbon. Yet, this modification can lead to other challenges, such as electrode stability. Nevertheless, it has been reported that stability can be resolved, similar to polymeric membranes [37]. Improving the biosensor's specificity is crucial to avoid false signals, especially in the presence of interfering molecules like uric acid, ascorbic acid, bilirubin, and certain drug compounds found in blood. Enhancing specificity remains a key focus area in biosensor development [167].

In addition to well-known traditional biomarkers, research has started to explore new biomarkers for DM diagnosis, including ATP, C-peptide, sorbitol, 1,5-anhydroglucitol, CRP, microRNA, and adiponectin [45]. Since DM is a complex disease with diverse factors affecting its onset, detecting multiple biomarkers from the same patient can help to increase the accuracy of detection and overcome weaknesses that may be present in each biomarker [205]. Moreover, investigating the interrelationship among different biomarkers can clarify disease dynamics and help distinguish between different types of DM. This novel biomarker detection method is still under-researched, including the use of carbon electrode-based electrochemical biosensors. Detection of specific biomarkers using this method can provide more information so that treatment can be personalized better for each individual. In addition, this method is also easy for medical practitioners to operate, enabling wider application in daily clinical and point-of-care practice.

Strict clinical validation is also important to ensure the accuracy of the biosensor in a clinical setting by being tested on diabetic patients. In addition, the demand for continuous monitoring is increasing, leading to the development of biosensors that can perform continuous monitoring of diabetes biomarkers. There is a need to integrate electrochemical biosensors using carbon-based electrodes with technologies such as the Internet of Things (IoT) and artificial intelligence (AI) to expand the potential of electrochemical biosensors for transferring real-time monitoring data to healthcare professionals.

However, there are several challenges to overcome, such as variations in the production process of disposable carbon electrodes, decreased stability over time, and loss of detection sensitivity for continuous use of carbon electrodes, as well as the ability to detect diabetes biomarkers directly in blood samples without the need for preparations such as dilution or complex extraction. Therefore, further research is needed to overcome these obstacles and ensure that electrochemical biosensors can become effective, accurate, and user-friendly tools in the diagnosis and management of diabetes mellitus.

## Conclusion

The global surge in diabetes cases has raised urgent concerns in public health, underscoring the need for more effective diagnostic tools. Enter electrochemical biosensors with carbon electrodes—a solution teeming with potential. A thorough examination of the use of these biosensors in detecting diabetes mellitus, focusing on pivotal biomarkers like glucose, HbA1c, GHSA, and insulin, provides invaluable insights. Various types of carbon-based electrodes, when modified with different materials, have been shown to enhance biosensor performance. The important role of aptamers, antibodies, and enzymes as bioreceptors play a key role in enabling specific and selective detection of diabetes biomarkers. Significant progress has been achieved in harnessing carbon electrode-based electrochemical biosensors for detecting HbA1c, GHSA, and insulin, with particular emphasis on glucose detection. This serves as a foundational framework for further exploration into detecting other biomarkers that can explain specific pathologies, such as ATP, C-peptide, 1,5-AG, sorbitol, CRP, microRNA, and adiponectin. Of the various types of carbon electrodes, GCE and SPCE are still the electrodes most popular among researchers, GCE is preferred for its high sensitivity, stability, and reproducibility, ensuring precise and accurate results, whereas SPCE is particularly promising for clinical applications due to its disposability, portability, cost-effectiveness, and high sensitivity despite its compact size. Overall, carbon-based electrochemical biosensors represent a promising avenue for developing point-of-care methods, aiming to improve the accuracy, efficiency, simplicity, and rapidity of diabetes diagnosis and disease management.

## Figures and Tables

**Table 1.** The comparison of the related reviews

Ref.	Year	Diabetes mellitus biomarker	Carbon-based electrode	Electrochemical biosensor	Biosensor for glucose	Biosensor for HbA1c	Biosensor for GHSA	Biosensor for insulin	Open research issue
[37]	2017	-	✓	-	✓	-	-	✓	✓
[36]	2023	-	✓	✓	✓	-	-	-	-
[38]	2022	✓	-	-	✓	✓	✓	✓	-
[39]	2015	-	-	✓	✓	✓	-	-	-
[40]	2022	✓	✓	✓	**-**	✓	✓	**-**	✓
[41]	2019	-	-	✓	✓	-	-	✓	✓
Our report		✓	✓	✓	✓	✓	✓	✓	✓

**Table 2.** Characteristics of traditional biomarkers diabetes mellitus.

Biomarkers	Duration of glycemia reflected	Normal range	Prediabetes range	Abnormal range	Advantages	Limitations	Ref.
Glucose	Real time^a^	70 to 99 mg/dL^a^ 4.4 to 6.6 mM^c^	100 to 125 mg/dL^a^	>126 mg/dL on fasting or >200 mg/dL on randomized test^a^ 7 mM ^c^	Direct measurement (reflects actual conditions)^b^ Common and easy to measure^b^	Need to fast^b^ Influenced by rapid lifestyle changes^b^ Less sensitive in the early stages^b^	^a^[2] ^b^[82] ^c^[83]
HbA1c	2-3 months^a^	<5.7 %^a^	5.7 to 6.4 %^a^	≥6.5 %^a^	Reflects 2-3 months of glucose control^d^ Patient does not need preparation^d^ Not affected by rapid lifestyle changes^d^	Sensitive to changes/disturbances in red blood cells^e^ Hemoglobin variants may give inaccurate results^e^ Blood samples must be intact^e^	^a^[2] ^d^[84] ^e^[85]
GHSA	2-3 weeks^f^	<13.35 %^f^	13.35 to 16.07 %^f^	≥16.07 %^f^	Patient do not need preparation^g^ Can be measured in serum or plasma^g^ Not affected by hematocrit changes^g^	Affected by altered albumin metabolism and thyroid dysfunction^g^ In obese patients, GHSA can be low^g^	^f^[66] ^g^[86]
Insulin	Produced when glucose is present^h^	25 mIU/L (0.86 ng/mL)^h^	-	-	Responds quickly to changes in glucose^g^ Information on insulin production^g^ Can provide insulin resistance information^g^	Individual variability^g^ May be affected by conditions other than diabetes^g^	^h^[73] ^g^[41]

**Table 3.** Comparison of carbon-based electrodes with other material-based electrodes.

Material	Advantages	Limitations	Ref.
Carbon	Low cost, flexibility, compatibility, and biocompatibility with various chemical or biological substances, good conductivity, inert, good cathodic potential range, ease of use and maintenance.	Less precision in some applications, prone to contamination, and low mechanical strength.	[24,112]
Gold	Biocompatible, stable in various conditions, high conductivity, larger cathodic potential range, high corrosion resistance.	Expensive, not suitable for some applications, reactive to some chemical compounds in biological samples	[112,113]
Platinum	High chemical stability, high conductivity, non-reactive with many compounds, high corrosion resistance.	Expensive, not suitable for all sensor applications requiring high precision	[114]
Titanium	Lightweight, high strength, corrosion resistance, biocompatibility.	Limited electrical conductivity, may require surface modification for certain sensor applications.	[115]
Silver	High electrical conductivity, antibacterial properties.	Prone to tarnishing, may react with sulfur compounds, not suitable for all environments.	[116,117]
Copper	Good electrical conductivity, relatively low cost.	Susceptible to oxidation, may not be suitable for long-term sensor applications without protective coatings.	[118]
Nickel	Corrosion resistant, good temperature stability.	Potential toxicity, not biocompatible, may not be suitable for biomedical sensor applications.	[119,120]

**Table 4.** Electrochemical biosensors for diabetes biomarkers using glassy carbon electrodes.

Biomarkers	Electrode modification	Redox probe	Detection methods	Results	Sample	Ref.
Glucose	NF-GA/MWCNTs-BSA-HFs--GOx/GCE	N_2_-saturated 50 mM PBS (pH 6.0)	CV	Linear range: 0.01-3.5 mM LOD: 17 μM Sensitivity: 167 μA/mM cm^2^ Storage stability: 120 days Recovery: 95.9 ~ 103.9 %	Human plasma	[125]
	PEI/PVA/GOx/GCE	50 mmol/L PB (pH 7.1)	DPV	LOD: 0.3 mmol/L Sensitivity: 11.79 μA/mM cm^2^ Recovery: 95.9 ~ 107.5 %	Human serum	[135]
	CHIT/GO/GOx/GCE	0.1 M PBS (pH 7.4)	CV	Linear range: 0.05-20 mM LOD: 0.02 μM Sensitivity: 1006.86 μA/mM cm^2^	D-glucose	[136]
	GOx/CoS-MWCNTs/NF/GCE	0.1 M PBS (pH 6.0)	CV	Linear range: 0.01-1.5 mM Sensitivity: 18.7 mA/M cm^2^ LOD: 0.005 mM	Human serum	[137]
	NF/GOx/IL/mPEG-MWCNTs/GCE	50 mM PBS (pH 7.0)	CV	Linear range: 2.0 to 0.95 mmol/L LOD: 0.2 μmolL	Human plasma	[138]
	CtCDH C291Y/GA/4-APh, 4-MBA/AuNPs/GC	50 mM TRIS buffer (pH 7.4)	CV	Linear range: 0.02-30 mM LOD: 6.2 μM Sensitivity: 3.1 μA/mM cm^2^ Stability: 90 % (20 days) Recovery: 95.0 ~ 97.4 %	Human saliva	[139]
HbA1c	Anti-HbA1c/rGO/GCE	[Fe(CN)_6_]^4−/3−^	CV/EIS	Linear range: 1 to 25 %	HbA1c standard	[127]
	Aptamer/Ru(bpy)_3_^2+^ @MPDA/ GCE	[Fe(CN)_6_]^4−/3−^	ECL	Linear range: 0.1 to 18.5 % LOD: 0.015 % Recovery: 99.82 ~ 102.69 % (human serum); 96.43 %-100.85 % (whole blood)	Human serum & whole blood	[129]
	PBA-PQQ/rGO/GCE	0.1 M PB (pH 8.0)	DPV	Detection range: 9.4 to 65.8 μg/mL LOD: 1.25 μg/mL	Human whole blood	[140]
GHSA	Aptamer/rGO-AuNP/GCE	[Fe(CN)_6_]^4−/3−^	SWV	Detection range: 2 to 10 μg/mL LOD: 0.07 μg/mL	Synthetic GHSA	[141]
	MIG/AuNP-OPPy/GCE	[Fe(CN)_6_]^4−/3−^	DPV	LOD: 1.2 mg/mL	Human serum	[142]
	MB-tDNA/Cu O-rGO/GCE_2_	[Fe(CN)_6_]^4−/3−^	DPV	Linear range: 0.02 to 1500 μg/mL LOD: 0.007 μg/mL	Human serum	[130]
Insulin	MB-Apt-AuNP//insulin/ /Apt/AuNP/GCE	[Fe(CN)_6_]^4−/3−^	DPV	Linear range: 0.1 pM to 1.0 μM LOD: 9.8 fM	Bovine Insulin	[131]
	Aptamer/cDNA/GA/ /MSTF/GCE	[Fe(CN)_6_]^4−/3−^	DPV	Linear range: 10-350 nM LOD: 3.0 nM Recovery: 96.28 to 108.05 %	Spiked serum	[143]
	Anti-insulin/CdS: Eu NCs-rGONRs/MWCNT/GCE	S_2_O_8_^2-^	ECL	Linear range: 0.0005 to 50 ng/mL LOD: 0.00040 ng/mL Recovery: 98.2 ~ 104 %	Insulin antigen, human serum	[144]
ATP	Apt/Fe_3_O_4_@Cu@Cu_2_O/ /GCE	[Ru(bpy)_2_(dppz)]^2+^	ECL	Linear range: 0.5 to 2500 nmol/L LOD: 0.17 nmol/L Recovery: 98.2 to 108 %	Human serum	[145]
	Apt/p-L-Glu/GCE	[Fe(CN)_6_]^4−/3−^	DPV	Linear range: 0.01 pM to 1.0 μM LOD: 0.01 pM	Human serum	[133]
MicroRNA	DNA/NSA/GCE	[Fe(CN)_6_]^4−/3−^	CV	Linear range: 10 nM-10 fM LOD: 20 fM	Urine	[146]
C-peptide	DNA/DA/GCE	Ru(bpy)_2_(mcbpy)^2+^	ECL	Linear range: 50 fg/mL to 16 ng/mL LOD: 16.7 fg/mL	Human serum	[134]

GOx: Glucose oxidase; NF: Nafion; GA: Glutaraldehyde; MWCNT: Multi walled carbon nanotube; BSA: Bovine serum albumin; HF: Hydroxy fullerene; PBS: Phosphate Bovine Serum; PEI: Poly(ethyleneimine); PVA: Poly(vinyl alcohol); CHIT: Chitosan; GO: Graphene oxide; CoS: Cobalt sulfide; IL: Ionic liquid; mPEG: Aminated polyethylene glycol; CtCDH C291Y: Corynascus thermophilus; 4-APh: 4-aminothiophenol; 4-MBA: 4-mercaptobenzoic acid; rGO: Reduced graphene oxide; MPDA: Mesoporous polydopamine; PBA-PQQ: Phenylboronic acid- Pyrroloquinoline quinine; MIG: Miglitol; OPPy; MB: Methylene blue; MSTF: Mesoporous silica thin-film; CdS:EU NCs: CdS nanoclusters loaded with europium(III); rGONR: Reduced graphene oxide nanoribbon; DA: Dopamine; CV: Cylic voltammetry; DPV: Differential pulse voltammetry; ECL: Electrochemiluminescence; EIS: Electrochemical impedance spectroscopy; SWV: Square-wave voltammetry.

**Table 5.** Electrochemical biosensors for diabetes biomarkers using screen-printed carbon electrodes.

Biomarkers	Electrode modification	Redox probe	Detection methods	Results	Sample	Ref.
Glucose	GOx/NF/MnO_2_-GNR/SPCE	0.1 M PB (pH 7.0)	CV	Linear range: 0.1 - 1.4 mM LOD: 50 μM Sensitivity: 56.32 μA/mM^1^ cm^2^	D-glucose	[162]
	GOx/Pt/rGO/P3ABA/SPCE	0.1 M PBS (pH 7.4)	Amp	Linear range: 0.25 - 6 mM LOD: 44.3 μM Sensitivity: 22.01 μA/mM cm^2^	D-glucose	[163]
	GOx/PtNP/PAA/SPCE	0.1 M PB (pH 7.0)	Amp	Linear range: 20 μM-2.3 mM LOD: 7.6 μM Sensitivity: 42.7 μA/mM cm^2^	D-glucose	[150]
HbA1c	SH-Aptamer/AuNP/SPCE	[Fe(CN)_6_]^4−/3−^	SWV	Linear range: 0.1-1000 ng/mL LOD: 0.2 ng/mL	Human whole blood	[154]
	anti-HbA1c/AuNP/SPCE	[Fe(CN)_6_]^4−/3−^	DPV/CV	Linear range: 20-200 μg/mL LOD: 15.5 μg/mL	HbA1c standard	[164]
	Aptamer/TBO/ /pTBA@MWCNT/SPCE	0.1 M PBS (pH 7.4)	Amp	Linear range: 0.006 - 0.74 μmol/L LOD: 3.7 nM	Human whole blood	[165]
	Anti-HbA1c/STV/SPCE-PET	[Fe(CN)_6_]^4−/3−^	Potentiometry	Linear range: 5.6 to 10.6%	Human whole blood	[166]
GHSA	Apt/GO-NHS/SPCE	[Fe(CN)_6_]^4−/3−^	SWV	Linear range: 0.001-10 mg/mL LOD: 0.031 μg/mL	Spiked serum	[167]
	SH-Apt/STV/SPCE	[Fe(CN)_6_]^4−/3−^	SWV	Linear range: 2×10^-6^ to 16 mg/mL LOD: 2.6 ng/mL Sensitivity: 10^-6^ to 1 mg/mL	Human plasma	[168]
	Apt/AuNI/SPCE	[Fe(CN)_6_]^4−/3−^	DPV	Linear range: 1-40 mg/mL	Mouse serum	[156]
	Fructosamine 6-kinase/SPCE	[Fe(CN)_6_]^4−/3−^	Amp	Linear range: 20 to 100 μM	Synthetic GHSA	[169]
	FAOx/SPCE	Ru(NH_3_)_6_^3+/2+^	CA	Linear range: 0.1 to 100 μg/mL LOD: 0.1 μg/mL	Human whole blood	[155]
	Aptamer/GO/SPCE	[Fe(CN)_6_]^4−/3−^	SWV	Linear range: 0.01 to 50 μg/mL LOD: 8.7 ng/mL	Human Serum	[170]
	Aptamer/GO-Pb/SPCE	[Fe(CN)_6_]^4−/3−^	SWV	Linear range: 0.001 to 10 μg/mL LOD: 0.77 μg/mL	Human serum	[171]
Insulin	Aptamer/Au/SPCE	0.1 M PBS	SWV	Linear range: 25 to 150 pM LOD: 18.5 pM Stability: 10 days (92 %)	Human whole blood	[172]
	Apt-MB/AuNP/SPCE	[Fe(CN)_6_]^4−/3−^	CV and SWV	Linear range: 0.05-15 nM LOD: 0.85 nM Stability: 30 days (87.52 %) Recovery: 92.0 ~ 98.8 %	Spiked human saliva	[173]
	apt-MB/ OMC-TPS/SPCE	0.1 M PB (pH 7.4)	DPV	Linear range: 1 fM to 10 pM LOD: 0.18 fM Stability: 21 days (92.8 %)	Human serum spiked	[29]
ATP	Apt/AuNP/SPCE	[Fe(CN)_6_]^4−/3−^	DPV	Linear range: 0.1 to 100 μM LOD: 7.43 μM LOQ: 24.78 μM	ATP standard	[152]
CRP	Anti-CRP/GQDs/SPCE	[Fe(CN)_6_]^4−/3−^	CV	Linear range: 0.5 to 10 ng/mL LOD: 0.036 ng mL^−1^ Sensitivity: 2.45 μA/ng mL cm^2^	Artificial serum	[159]
microRNA	DNA-ANSAm/SPCE	[Fe(CN)_6_]^4−/3−^	CV	Linear range: 10^-9^-10^-14^ M LOD: 17 fM	Urine	[160]
1,5-AG	PROD/PT-rGO-PtPd/SPCE	[Fe(CN)_6_]^4−/3−^	DPV	Linear range: 0.1-2.0 mg/mL LOD: 30.0 μg/mL Recovery: 99.8 to 106.8 %	Human serum	[161]

GOx: Glucose oxidase; NF: Nafion; MWCNT: Multi-walled carbon nanotube; PBS: Phosphate Bovine Serum; GO: Graphene oxide; rGO: Reduced graphene oxide; GNR: Graphene nanoribbons; Pt: Platinum; P3ABA: Poly(3-aminobenzoic acid; TBO: Toluidine blue O; pTBA: Poly(2,2ʹ:5ʹ,5″-terthiophene-3ʹ-p-benzoic acid); PET: Polyethylene terephthalate; AuNI: Gold nanoisland; FAOx: Fructosyl amino acid oxidase; OMC: Ordered mesoporous carbon; TPS: 1,3,6,8-pyrenetetrasulfonate; GQDs: Graphene quantum dots; PROD: Pyranose oxidase; PtPd: Platinum-Palladium; CV: Cylic voltammetry; DPV: Differential pulse voltammetry; SWV: Square-wave voltammetry. Amp: amperometry.

**Table 6.** Electrochemical biosensors for diabetes biomarkers using other carbon-based electrodes.

Biomarker	Electrode	Electrode modification	Redox probe	Detection method	Results	Sample	Ref.
Glucose	CPE	GOx/PAA	0.1 M PBS (pH 7.0)	CV	Linear range: 0.05-1.0 mM LOD: 69 μM Sensitivity: 5.3 μA/mM cm^2^	D-glucose	[176]
		GOx/Fc-graphite/CA	0.5 M NaCl	Amp	Linear range: 0.05-3.00 mM LOD: 0.0240 mM Sensitivity: 0.290 μA/mM	D-glucose	[195]
		GOx-NiFe_2_O_4_/AC	0.1 M PBS (pH 7.0)	CV	Linear range: 2-10 mM LOD: 1.1 mM LOQ: 3.7 mM	Blood plasma	[28]
		GOx/Se-MCM-41	0.05 M PB (pH 7.0)	CV	Linear range: 0.01 to 2 mM LOD: 0.1 mM *K*_m_: 0.02 mM Stability: 91 % (10 days)	D-glucose	[196]
		GOx/SiO_2_/lignin	0.1 M PBS (pH 7.0)	CV and CA	Linear range: 0.5-9 mM LOD: 145 μM Sensitivity: 0.78 μA/mM	Glucose infusion solution	[197]
		Fc/GOx/SBA-15	0.2 M PBS (pH 7.0)	Amp	Linear range: 2.0-18.2 mM Sensitivity: 1.5 μA/cm^2^ mM	D-glucose	[198]
		GOx/ZnO	0.1 M PBS (pH 7.0)	CV	Linear range: 40 to 380 μM LOD: 8 μM	D-glucose	[199]
	BDD electrode	GOx/AuNP	0.1 M PBS (pH 7.0)	CV	Linear range: 0.1 to 0.9 M	D-glucose	[180]
		Nafion/GOx/ /APTES	0.01 M PBS (pH 7.0)	Amp	Linear range: 35 μM to 8.0 mM LOD: 30 μM	D-glucose; human serum	[200]
		NAD-GDH/DT-D	0.1 mM Menadione	CV	LOD: 20 μM & 3 μM	D-glucose; human serum	[181]
	Graphene electrode	GOx/CHIT/LIGE	0.05 M PBS (pH 7.0)	CA	Linear range: 0-8 mM LOD: 0.431 mM Sensitivity: 1.39 to 1.81 μA/μM cm^2^ Stability: 90 % (10 days); 72-85 % (>10 days)	D-glucose	[201]
	Graphene electrode	GOx/CHIT/ /PB/LIGE	0.1 M PBS (pH 7.4)	CV	Linear range: 25 to 300 μM LOD: 9.6 μM Sensitivity: 457 nA/ μM cm^2^	D-glucose & artificial sweat	[202]
		GOx-BSA/Fc/LIGE	0.01 M PBS (pH 7.4)	Amp	Linear range: 0-11 mM LOD: 0.04 μM Sensitivity: 11.3 μA/μM cm^2^	Human serum	[187]
		GOx/PtNP/ /acetic acid-LIGE	0.1 M PBS (pH 7.4)	Amp	Linear range: 0.0003-2.1 mM LOD: 0.3 μM Sensitivity: 65.6 μA/μM cm^2^	D-glucose & sweat	[203]
		GOx/pyNHS/LSGE	PBS	Amp	Linear range: 0.04 to 4.0 mM LOD: 19.8 μM Sensitivity: 16.35 μA/μM cm^2^	D-glucose	[186]
	Graphite electrode	GOx/AuNP/ /GO/GFME	N_2_ -saturated PBS (pH 7.0)	Amp	LOD: 1.2 μM Stability: 90.22 % (10 days)	D-glucose	[188]
		Ppy/GOx/ /AuNPs/GRE	6 mmol/L phenazine methosulfate	Amp	Linear range: 0.99 to 19.9 mmol/L LOD: 0.2 mmol/L Sensitivity: 21.7 μA/μM cm^2^	D-glucose; human serum	[204]
		GA-GOx/SAM/ /DGNs/GRE	6.0 mM Phenazine methosulfate	Amp	Linear range: 0.1 to 10 mM LOD: 0.019 mM Stability: 73.25 % (12 days)	D-glucose, Human serum	[189]
		GOx/AuNP/ /p(EDOTBN)/PGE	0.1 M PBS (pH 6.5)	CV	Linear rang: 0.0.169 to 10 mM LOD: 0.008461 mM Sensitivity: 38.365 μA/μM cm^2^ Stability: 4 weeks	D-glucose	[190]
HbA1c	Graphite electrode	Apt/rGO-Au/GS	[Fe(CN)_6_]^4−/3−^	DPV	Linear range: 1 nM to 13.83 μM LOD: 1 nM Sensitivity: 269.2 μA/cm^2^ Recovery: 94.0 to 109.5 %	Human whole blood	[191]
Insulin	Graphene electrode	MB-Apt-AuNP/ /insulin/Apt/ /AuNP/LSGE	[Fe(CN)_6_]^4−/3−^	DPV	Linear range: 0.1 pM to 1.0 μM LOD: 22.7 fM Stability: 90.93 % (20 days)	Bovine Insulin	[131]

GOx: Glucose oxidase; PAA: Poly L-aspartic acid; Fc: Ferrocene; SE-MCM-41: Mesoporous silica: Mobil Composition of Matter No. 41; SBA-15: Mesoporous silica: Santa Barbara Amorphous-15; NAD-GDH; Nicotinamide adenine dinucleotide-Glucose dehydrogenase; DT-D: DT-diaphorase; CHIT: Chitosan; LIGE: Laser-induced graphene electrode; BSA: Bovine serum albumin; pyNHS: pyrenebutyric acid N-hydroxysuccinimide ester; LSGE: Laser-scribed graphene electrode; GFME: Graphite fiber microelectrode; Ppy: Oxidized polypyrrole; GRE: Graphite rods electrode; SAM: Self-assembly monolayer; PGE: Pencil graphite electrodes; p(EDOTBN): Polymer (4-(dihexylamino)-9,12-bis(2,3-dihydrothieno[3,4-b][1,4]dioxine-5-yl)-7H-benzo[de]benzo[4,5]imidazo[2,1-a]izoknolin-7-one); GS: Graphite sheets; MB: Methylene blue; PtNP: Platinum nanoparticle; CV: Cylic voltammetry; DPV: Diffential pulse voltammetry; Amp: amperometry; CA: Chronoamperometry.

## List of abbreviations

1,5-AG: 1,5-anhydroglucitol
AA: Ascorbic acid
AGE: Advanced glycation end
Anti-HbA1c: Antibody HbA1c
AuNPs: Gold nanoparticles
Apt: Aptamer
ADP: Adenosine diphosphate
AMP: Adenosine monophosphate
ATP: Adenosine triphospate
BDD: Boron-doped diamond
BSA: Bovine serum albumin
CA: Chronoamperometry
CHIT: Chitosan
CPE: Carbon paste electrode
CRP: C-Reactive Protein
CTP: Cytidine triphosphate
CV: Cyclic voltammetry
CVD: Chemical vapor deposition
DGN: Dendritic gold nanostructures
DKD: Diabetic kidney disease
DM: Diabetes mellitus
DNA: Deoxyribonucleoic acid
DPV: Diffential pulse voltammetry
ECL: Electrochemiluminescence
EDC: 1-ethyl-3-(3-dimethylaminopropyl) carbodiimide
EIS: Electrochemical impedance spectroscopy
FAD: Flavin adenine dinucleotide
FAOx: Fructosyl amino acid oxidase
GA: Glutaraldehyde
GCE: Glassy carbon electrode
GDH: Glucose dehydrogenase
GFE: Graphite fiber microelectrodes
GFME: Graphite fiber microelectrode
GHSA: Glycated human serum albumin
GO: Graphene oxide
GOx: Glucose oxidase
GTP: Guanosine triphosphate
HbA1c: Glycated hemoglobin
HSA: Human serum albumin
Km: Michaelis-Menten constant
LIG: Laser-induced graphene
LOD: Limit of detection
LSG: Laser-scribed graphene
MB: Methylene blue
MWCNT: Multi walled carbon nanotube
NAD: Nicotinamide adenine dinucleotide
NF: Nafion
NHS: N-hydroxysuccinimide
PGE: Pencil graphite electrodes
PtNP: Platinum nanoparticles
rGO: Reduced graphene oxide
SAM: Self-assembly monolayer
SPCE: Screen printed carbon electrode
SWV: Square-wave voltammetry
UA: Uric acid
UTP: Uridine triphosphate
ε-FK: ε-fructosyl lysine
